# Investigation of the Physicochemical Properties of Vegetable Oils Blended with Sesame Oil and Their Oxidative Stability during Frying

**DOI:** 10.1155/2022/3165512

**Published:** 2022-03-30

**Authors:** Fereshteh Ramroudi, Seyed Ali Yasini Ardakani, Arefeh Dehghani-tafti, Elham Khalili Sadrabad

**Affiliations:** ^1^Zoonotic Diseases Research Center, Department of Food Hygiene and Safety, School of Public Health, Shahid Sadoughi University of Medical Sciences, Yazd, Iran; ^2^Department of Food Science and Technology, Yazd Branch, Islamic Azad University, Yazd, Iran; ^3^Department of Biostatistics, School of Public Health, Shahid Sadoughi University of Medical Sciences, Yazd, Iran; ^4^Nutrition and Food Security Research Center, School of Public Health, Shahid Sadoughi University of Medical Sciences, Yazd, Iran

## Abstract

To investigate the antioxidant activity and physicochemical properties of oil, sunflower (SFO) and corn oil (CO) and their combinations with sesame oil (SO) were prepared. The analyses of fatty acid composition (GC-FID), oxidative stability index (Rancimat), smoke point, and antioxidant activity (DPPH) were done on oil samples. Then, the frying process in presence of potato chips was done for 3 days at 180°C. Oil samples were gathered after each frying cycle and chemical analysis (peroxide value, free fatty acid, p-anisidine value, TOTOX, total polar content, TBARS, and conjugated diene and triene) was measured. The major fatty acid composition of oil samples was linoleic acid, oleic acid, palmitic acid, and stearic acid. The OSI of oil samples was reported as CSO > SSO > SO > CO > SFO. The smoke point of all samples was in the standard limit. The SFO with 266.50°C had the highest smoke point. The antioxidant activity of samples was reported as SO > CSO > CO > SSO > SFO. The IC50 of SO was 52.17 mg/g which was higher than other samples. The result of frying indicated that prolonged heating process would increase the thermal oxidation. It was shown that oils blended with SO had good stability during deep frying. Therefore, blending oil with SO is considered as an economic approach to improve the oil oxidation stability.

## 1. Introduction

Deep fat frying is defined as the dynamic process that food pieces immersed in large quantity of heated vegetable oils. In the frying process along with high temperature, the food water was evaporated and released in the surrounding oil [1]. Vegetable oils are considered as an ideal cooking medium due to their health beneficial effects. Although exposure to oxygen, heat, and light enhance the oil deterioration and reduce the nutritional value of oil [2]. Different reactions such as thermal oxidation, polymerization, and hydrolysis in frying oil could deteriorate the quality of oil and have adverse effects on flavor and odor of oil and fried foods [3]. These reactions lead to production of volatile and nonvolatile compounds which cause changes in sensory, nutritional, and functional quality of fried oils [4]. In addition, the nonvolatile compounds which remain in oils could be absorbed by food and promote the oil degradation [5, 6]. The presence of food in frying process can accelerate the oil degradation [3]. Although, the continuous use of the degraded frying oils could be harmful to health and cause atherosclerosis, liver damage, and intestinal tumors [3, 7].

Along with the effects of frying conditions (heating temperature, frying time, and volume) on quality of oil and food, the type of oil and food is considered important in degradation of fried oil [8]. The blending of different oils is an important and simple method to get a healthy oil mixture with desired oxidative properties [9, 10]. The mixing of vegetable oils enhances the nutritional and physical properties of oils as well as chemical properties such as oxidative stability [10]. Sesame oil with 85% unsaturated fatty acid is introduced as stable oil against oxidation, due to the presence of lignans and tocopherols [11]. The use of sesame oil in frying is not economical; therefore, its mixture with other vegetable oils can reduce the cost and be a good alternative of traditional oils [10]. Due to the popularity of sesame oil by Iranian consumers, the combination of sesame oil with other vegetable oils had increased its consumption in recent years. Although great efforts has gone into investigation of vegetable oil combination and their chemical composition, less attention has been given to examining the use of sesame oil. On the other hand, by investigating the chemical quality of sesame oil, its probable application in combination with other vegetable oil to extend the shelf life of oil as well as increasing the nutritional value could be used. In the current study, the performance of sunflower oil, corn oil, and sesame oil and their combination with sesame oil during actual frying of potato chips were studied.

## 2. Material and Method

### 2.1. Reagents

Sodium thiosulfate, acetic acid, chloroform, potassium iodide, starch, trichloroacetic acid, 2-thiobarbituric acid, hydrochloric acid, p-anisidine, isooctane, 2,2-diphenyl-1-picrylhydrazyl, methanol, 1,1,3,3-tetraethoxypropane, and n-hexane were purchased from Merck, Germany. The refined sunflower oil (SFO), corn oil (CO), and sesame oil (SO) were purchased from the local company of Yazd, Iran. Fresh potato was purchased from local market of Yazd, Iran.

### 2.2. Oil Preparation

The combinations of sunflower/sesame oil (SSO) and corn/sesame oil (CSO) were prepared in the proportion of 3/2 w/w. This proportion was selected due to industrial oil production of Iran. The oil samples were stored in the cold condition (4°C) until the experiments were done. All experiments were done in triplicate.

### 2.3. Initial Characteristics of Unheated Oil Samples

#### 2.3.1. Fatty Acid Composition by GC-FID

The gas chromatography–flame ionization detection (GC-FID, Yang Lin 6500, South Korea) with capillary column (120 m × 2.5 mm i.d.; 0.25 *μ*m) was used to determine the fatty acids composition of the samples. The detector temperature was programmed from 90°C to 240°C (seven minutes at 90°C; ten minutes at 150°C; 15 minutes at 200°C; and 20 minutes at 240°C) and held at 240°C for 50 minutes. Helium gas (99.99%) was used as the carrier at 20 ml/min, and hydrogen and air at a ratio of 1 : 30 were used as oxidant. The retention time of methyl ester of samples was compared with standard [12]. The following equation was used to calculate the oxidizability value (COX) of oil samples according to percentage of unsaturated C18 fatty acids [13]:

(1) $$\begin{array}{c} \text{COX value}=\frac{\left[1\times \left(\text{C}18:1\%\right)+10.3\times \left(\text{C}18:2\%\right)+21.6\times \left(\text{C}18:3\right)\right]}{100}. \end{array}$$

#### 2.3.2. Antioxidant Activity by DPPH

The DPPH solution was prepared by the addition of 24 mg DPPH in 100 methanol which was diluted to reach the absorbance of 0.98 ± 0.02 at 517 nm (working solution). Then, 2.5 ml of working solution was added to 500 *μ*l diluted oil with isooctane. The radical scavenging activity (RSD) was measured by the following equation:

(2) $$\begin{array}{c} \%\text{RSD}=\left[\frac{\left(\text{Ac}–\text{As}\right)}{\text{Ac}}\right]\times 100, \end{array}$$

where the Ac and As are the absorbance of control (contains 500 *μ*l isooctane instead of sample) and sample, respectively.

#### 2.3.3. Oxidative Stability Index (OSI)

In order to measure oxidative ability, the Metrohm Rancimat apparatus (model 743, Herisau, Switzerland) was used. Approximately, 3 g oil was weighed into the reaction vessels, and 70 ml deionized water was added to the conductometric cells. Then, the clean, filtered, and dried air was bubbled through the hot oil (110°C) with the rate of 20 l/h [14].

#### 2.3.4. Smoke Point

The smoke point was measured according to the AOCS Method Cc 9a-48. The oil samples were poured into cup and heated up to 40-50°C/min. The smoke point was determined when the thin and continuous bluish smokes were seen [15].

### 2.4. Frying Process

Potatoes were peeled and sliced to reach the thickness of 2 mm and kept in distilled water until frying. The frying process was done according to Alireza et al. study with some modifications [9]. A proportion of 100 g dried potato chips were fired in 3.5 L oil with the temperature of 180°C for 2.5 min with interval of 17.5 min for a period of 3.5 h per each day during three days. At the end of each day, the hid of fryer was put and allowed the oil to be cooled overnight. The oil samples were collected after each day frying for further analysis [9]. This process was done for each oil samples in triplicate.

### 2.5. Analysis of Fried Oils

The oil samples were gathered after each frying cycle and chemical analysis (peroxide value, free fatty acid, p-anisidine value, TOTOX, total polar content, TBARS, and conjugated diene and triene) was measured.

#### 2.5.1. The Peroxide Value (PV)

Oil samples (5 g) were mixed with acetic acid-chloroform (30 ml) in ratio of 3 to 2. After the addition of 0.5 ml potassium iodide, samples were stored at dark room for 1 min. Then, samples were titrated with sodium thiosulfate solution in presence of starch solution until the blue color has disappeared. The PV was expressed as meq oxygen/kg oil [16].

#### 2.5.2. p-Anisidine Value (p-AV)

The p-anisidine value was determined according to AOCS official method Cd 18-90 method [17].

#### 2.5.3. TOTOX Value

The total oxidation or TOTOX value is calculated as TOTOX = 2PV + p − AV [18].

#### 2.5.4. Total Polar Content (TPC) and Free Fatty Acid (FFA)

The TPC and AV (%) were evaluated by TestoTM270 cooking oil tester. The TPC concentration of oil samples would be determined by the changes in dielectric constant of oil.

#### 2.5.5. Thiobarbituric Acid-Reactive Substances (TBARS)

2 ml of TBA reagent (15 g trichloroacetic acid, 0.375 g thiobarbituric acid, 2 ml HCl, and 82.9 ml distilled water) was added to 200 *μ*l oil samples and 800 *μ*l distilled water. The absorbance of centrifuged samples was recorded against blank at 532 nm. The results were expressed as mg MDA/kg oil according to standard curve of 1,1,3,3-tetraethoxypropane.

#### 2.5.6. Conjugated Diene (CD) and Triene (CT)

Briefly, 0.03 g of oil sample was dissolved in 25 ml Isooctane. The absorbance of samples was read at 233 and 268 nm for conjugated diene and triene, respectively [19].

### 2.6. Statistical Analysis

All of the analyses were carried out in triplicate and reported as the mean ± SD. The significant differences (*p* < 0.05) between the means were determined by Duncan's multiple-range test. The data analysis was performed using SPSS version 16.0.

## 3. Results and Discussion

### 3.1. Fatty Acid Composition

The presence of fats in human dietary is considered important due to providing energy, nutrients, carbohydrates, proteins, and vitamins [19, 20]. Therefore, use of healthy vegetables oils which could meet the human dietary needs seems essential. The blending of vegetable oils has been introduced as the simple and acceptable way to produce the oil with desired textural and oxidative properties [10]. Therefore, in the current study, the sesame oil with high nutritional value was used in combination with other oils to determine the oxidative properties during deep fat frying. In other words, the selection of corn and sunflower oils was made due to their common use as cooking media. The results of fatty acid composition in oil samples are given in Table 1. As it is obvious, the predominant fatty acids in all oil samples were estimated linoleic acid, oleic acid, palmitic acid, and stearic acid which is in consistence with results of Ghosh et al. [21], Farhoosh et al. [22], and Tan et al. [9]. The presence of high oleic acid and total monounsaturated fatty acids in oils might have protective role in cardiovascular diseases [23]. According to fatty acid composition, the sunflower oil with high MUFA might be more stable to oxidation during deep fat frying. However, the minor components, positional distribution of the glycerol backbone of fatty acids, and type of antioxidants could be considered important for oil susceptibility to oxidation [23]. It was mentioned that differences in the composition of oleic and linoleic fatty acids could cause some changes in oil composition. Oil samples with linoleic fatty acids higher than 50% are considered as polyunsaturated oils [24]. In current study, linoleic fatty acids of SSO samples with 54.55% were determined higher than other oils. Owing to high amount of palmitic acid in CO (12.5%) and CSO (12.45%), the SFA of these oils was estimated higher than other samples. The PUFA/SFA ratio is used to measure the amount of polyunsaturation of oils. In the current study, the greatest and lowest PUFA/SFA ratios with significant differences were reported in SO (5.23%) and CSO (2.73%), respectively. As can be seen, the blending of oils with sesame oils led to decrease in PUFA/SFA ration. The SSO had higher PUFA content, which make it sensitive to thermal oxidation. In general, the CSO had higher SFA which is considered more stable to oxidation. The high MUFA/PUFA ratio indicated the thermal oxidative stability of oils during frying [22]. Although, the lower MUFA/PUFA ratio was determined as higher nutritive value due to presence of essential fatty acids. Consequently, SSO had the lowest amount of MUFA/PUFA ratio. It was shown that blending oils prevent sharp decrease in unsaturated fatty acids, so it cause higher stability of oils during frying [9].

### 3.2. Oxidative Stability Index (OSI)

The results of OSI oil samples are given in Table 2. The differences in the oxidative stability of oils depend on different factors such as the chemical composition of initial raw material, mechanical damages, preliminary humidity and moisture of seeds, presence of contaminants, degree of seed maturity, and oil processing conditions [25]. In the current study, the blended oil samples showed higher OSI. The OSI of oil samples was reported as CSO > SSO > SO > CO > SFO. Oxidative stability index (OSI) of oil samples contain high amount of PUFA could increase by blending with high MUFA oils [26] which is in agreement with current results. Therefore, it could be concluded that oxidative stability of blended oils is related to PUFA and MUFA content [26, 27].

### 3.3. Smoke Point

The smoke point indicates the breakdown of fats to glycerol and FFAs [28]. As shown in Table 2, the smoke point of all samples was higher than standard limit (170°C). The lowest and highest smoke points were measured for SO (242.5°C) and SFO (266.5°C), respectively. In fact, the addition of SFO to SO caused the increase in smoke point. It was shown that smoke point is depended on FFA content and refinement process. Essien et al. reported the smoke point of 184.86°C for sesame oil [29] which is lower than current results. In general, the high smoke point oils have better thermal stability during frying [30].

### 3.4. Antioxidant Activity

Due to differences in the phenolic content of oil, the antioxidant activity of oils is varied [31]. The antioxidant activity of samples was reported as SO > CSO > CO > SSO > SFO. The high antioxidant activity of sesame oil and its blend with corn oil demonstrating interaction of their antioxidants (Table 2). The results showed that blended oils were more stable to oxidation which is in agreement with results of Boukandoul et al. [32], Bhatnagar et al. [26], and Abdel-Razek [33]. The blending of oils would increase the natural antioxidants, MUFA, and PUFA which could increase the ability of samples to scavenge DPPH [26].

### 3.5. Oil Changes during Deep Fat

Different parameters are needed to investigate the series of chemical reactions during frying [34]. Due to the limited validity of oxidation methods and food matrix, the combination of different detection methods is recommended [35]. Therefore, in order to monitor the oxidation of oil samples during deep frying, the PV, p-AV, FFA, TOTOX, TPC, TBARs, and conjugated diene and triene were measured (Table 3). Peroxide value and free fatty acid are the two of the most common parameters used for monitoring the edible oil quality [31]. The acid value indicates the free fatty acids (FFA) produced due to hydrolysis in a triacylglycerol [23]. Therefore, the FFAs promote the oil fuming and off flavor and then accelerate oil oxidation. Fatty acid oxidation forms hydroperoxides which is measured by PV. In order to evaluate the degree of oxidative degradation in oils, the PV and p-AV are commonly used [23]. During deep frying, the oil degradation could be affected by heat and mass transfer which occurred by the nature of fried foods [2]. The frying of foods in presence of air and water initiates a series of interrelated reactions [2]. It was shown that the moisture and fat content and thermal conductivity of fried food are the important factors in oil deterioration [2]. According to Codex Alimentarius, the maximum permitted peroxide value is set as 10 meq oxygen/kg for vegetable oil [31]. The PV increased during 3 days frying in the order: SSO > SFO > SO > CSO > CO. Except for CO and CSO, all oil samples had PV higher than permissible amount after second day. It is obvious from the results that by increasing in the frying time and continuous exposure to air and light, the primary oxidation products (PV) have increased. Tekin et al. investigated the stability of hazel nut, olive pomace, grape seed, and sunflower oil during frying. It was shown that olive pomace and hazel nut have better thermal stability in comparison with other oils [36]. On the other hand, high contents of linoleic acid in SSO samples could increase the oxidation susceptibility [37].

The p-AV of fresh oil samples varied from 5.69 to 10.05. The higher amount of aldehydes in fresh oils is attributed to the preservation of aldehydes during partial refining and cannot be considered as oxidation markers [32]. The results of p-AV of different oil samples were varied during three days of frying. Although the blended oil samples (CSO and SSO) were shown the lowest p-AV in comparison to other oils during three days of frying. The highest p-AV was measured in SFO with 137.26 in the last day of frying. The highest increase was observed in the first and second days of frying. These differences could be attributed to chemical; structure, oil composition, processing condition, and presence of metal ions in various oil samples [2, 32].

During the frying process, the highest TOTOX was reported in SFO, SO, and their combination which is due to the high amount of PUFA in these oils. The lowest TOTOX value was measured in corn oil and combination with sesame oil, which indicated the higher stability to oxidative rancidity.

The TPC of oils demonstrates the oxidation and hydrolysis which is taken place in the oil [4]. Different researches suggested the threshold level of 25-27% for TPC [4]. In our study, TPC of all samples was considered lower than permitted level which is in consistence with Matthaus [38], Santos et al. [39], and Ali et al. [18]. Although Tekin et al. reported the increase in TPC of fried oil samples after 3 days higher than permitted level [36]. The TPC of fresh oil samples was ranged from 6.08 to 6.75%. After three days frying, the increase in TPC of oils was recorded in order of SFO > SSO > CO > CSO > SO. The higher TPC of SFO and its combination are due to higher degree of unsaturation. As it is shown, the sesame oil and its blending with corn had effectively lower TPC after 3 days of frying. In Boukandoul et al.'s study, the effectiveness of blended oil during frying has been proved [32].

Conjugated dienes (CD) produce during polyunsaturated fatty acids oxidation and thermal formation of oligomers and polymers [36]. In the other word, oxidation could be resulted in alteration of double bonds of unsaturated fatty acids [39]. The CD of all samples was increased through frying days. The highest CD was shown in SSO (16.89%) after 3 days frying. The presence of high levels of PUFA in SSO (54.77%) will effect on the higher production of conjugated dienes during thermal treatment which is in agreement with Tekin et al.'s results [36]. In general, the current results clearly revealed that after three days of frying, all oil samples had oxidation levels above the recommended levels which are in agreement with results of Song et al. [35]. It was shown that the highest and lowest conjugated triene values (CT) were reported in SSO and SO, respectively. The increase order of TC in oil samples was as SSO > SFO > CO > CSO > SO. It could be said that due to low percentage of MUFA in SSO, the TC value is higher than other oils, which indicate lower oxidative stability [17]. In addition, the high content of CT value in SSO could be related to higher concentration of polyunsaturated fatty acids (PUFA) [37]. Almoselhy et al. showed higher CT value in sunflower which was attributed to higher amount of activated methylene groups in PUFA [40]. It was shown by researchers that formations of conjugated dienes are faster than conjugated trienes [16, 17], which is in acceptance with our results.

It should be effective to mention that oxidative stability of vegetable oil is affected by different factors including oil type, presence of metals as pro-oxidants (Fe and Cu), antioxidant types and concentrations, degree of unsaturated fatty acids, action of enzymes, and environmental conditions (light, oxygen, temperature, and production conditions) [37, 40, 41]. In general, SO showed the highest oxidative stability among unblended oils which was due to lower PUFA content. Therefore, its blending with other vegetable oils especially corn oil improves the thermal stability. Due to presence of natural antioxidant in SO, the antioxidant activity of blended oil could be improved.

## 4. Conclusion

In current study, the thermal oxidation was increased during three days of frying. It was shown that all chemical parameters increased in all oil samples. Although our results indicated that blending of oils could be effective way to retard oxidation and safety of oils during deep frying. In the current study, the oils blended with sesame oil presented good stability during deep frying. In other words, blending oil with sesame oil is considered as an economic approach to improve the oil oxidation stability. Among blended oil, combination of corn oil with sesame oil showed better function in terms of nutritional value and oxidative stability. Sesame oil with high antioxidant activity could be a beneficial to decrease the application of synthetic antioxidant in oil. Therefore, due to complex chemical reactions during frying, use of combination methods to monitor thermal oxidation is appropriate. The high degradation rate of frying oils raise concerns about the safety of used oils in restaurants.

## Figures and Tables

**Table 1:** Fatty acid composition of oil samples.

Fatty acid composition	CO	SFO	SO	CSO	SSO
Palmitic acid (C_16_:0)	12.50 ± 0.14^a^	6.40 ± 0.14^b^	10.30 ± 0.14^c^	12.45 ± 0.49^a^	9.75 ± 0.07^c^
Palmitoleic acid (C_16_:1)	0.10 ± 0^a^	0.05 ± 0.06^a^	0.05 ± 0.06^a^	0.10 ± 0^a^	0.19 ± 0.01^a^
Stearic acid (C_18_:0)	2.45 ± 0.21^a^	2.45 ± 0.21^a^	5.05 ± 0.07^b^	3.80 ± 0.14^c^	4.77 ± 0.10^b^
Oleic acid (C_18_:1)	33.30 ± 0.14^a^	40.05 ± 0.06^b^	38.35 ± 0.77^c^	36.20 ± 0.42^d^	29.30 ± 0.14^e^
Linoleic acid(C_18_:2)	48.15 ± 0.07^a^	49.10 ± 0.14^b^	44.55 ± 0.07^c^	45.35 ± 0.07^d^	54.55 ± 0.07^e^
Linolenic acid (C_18_:3)	1.25 ± 0.07^a^	0.62 ± 0.10^b^	0.32 ± 0.03^cd^	0.55 ± 0.07^bc^	0.22 ± 0.03^d^
Arachidic acid (C_20_:0)	0.45 ± 0.07^a^	0.22 ± 0.03^b^	0.40 ± 0^a^	0.20 ± 0^b^	0.31 ± 0.01^ab^
Gondoic acid (C_20_:1)	0.31 ± 0.01^a^	0.21 ± 0.01^b^	0.10 ± 0^c^	0.20 ± 0^b^	0.12 ± 0.02^c^
Behenic acid (C_22_:0)	0.32 ± 0.03^a^	0.32 ± 0.03^a^	0.10 ± 0^b^	0.21 ± 0.01^c^	0.50 ± 0^d^
Lingoceric acid (C_24_:0)	0.40 ± 0^a^	0.11 ± 0.01^b^	0.11 ± 0.01^b^	0.11 ± 0.01^b^	0.21 ± 0.01^c^
SFA	16.13 ± 0.38^a^	9.51 ± 0.26^b^	15.97 ± 0.18^a^	16.77 ± 0.60^a^	15.55 ± 0.07^a^
MUFA	33.71 ± 0.12^a^	40.32 ± 0.11^b^	38.51 ± 0.83^c^	36.50 ± 0.41^d^	29.61 ± 0.12^e^
PUFA	49.40 ± 0.14^a^	49.72 ± 0.24^a^	44.87 ± 0.03^b^	45.90 ± 0.14^c^	54.77 ± 0.03^d^
PUFA/SFA	3.06 ± 0.06^a^	5.23 ± 0.12^b^	2.81 ± 0.03^ac^	2.73 ± 0.08^c^	3.52 ± 0.01^d^
MUFA/PUFA	0.68 ± 0^a^	0.81 ± 0^b^	0.85 ± 0.01^c^	0.79 ± 0^b^	0.54 ± 0^d^
COX value	5.56 ± 0.02^a^	5.59 ± 0.03^a^	5.04 ± 0^b^	5.15 ± 0.02^c^	5.96 ± 0^d^

CO: corn oil; SFO: sunflower oil; SO: sesame oil; CSO: sesame-corn oil; SSO: sunflower-sesame oil. Different letters in each row show significant differences at level of *p* < 0.05.

**Table 2:** Chemical characteristics of oil samples.

Oil type test	Oxidative stability index (h)	Smoke point (°C)	DPPH (IC_50_, mg/g)
CO	16 : 47 ± 0 : 03^a^	257.50 ± 3.536^ac^	162.35 ± 2.61^a^
SFO	14 : 36 ± 0 : 02^b^	266.50 ± 2.121^a^	305.39 ± 7.45^b^
SO	18 : 31 ± 0 : 01^c^	242.50 ± 3.536^b^	52.17 ± 1.38^c^
CSO	22 : 12 ± 0 : 03^d^	249 ± 1.414^bc^	81.27 ± 1.47^d^
SSO	20 : 01 ± 0 : 02^e^	259 ± 1.414^ac^	222.05 ± 1.93^e^

CO: corn oil; SFO: sunflower oil; SO: sesame oil; CSO: corn oil-sesame; SSO: sunflower-sesame oil. Different letters in each column show significant differences at level of *p* < 0.05.

**Table 3:** Oxidative parameters of oil samples during 3 days of frying.

Analyses	Oil samples	Before deep frying	Day 1	Day 2	Day 3
PV	CO	0.82 ± 0.05^A,a^	2.50 ± 0.36^A,b^	2.80 ± 0.31^A,c^	5.24 ± 1.97^A,d^
	SFO	0.36 ± 0.05^B,a^	11.12 ± 4.64^BC,b^	12.21 ± 4.38^B,c^	14.54 ± 3.67^BC,d^
	SO	0.49 ± 0^C,a^	8.34 ± 0.08^CD,b^	10.90 ± 0.16^BC,c^	12.76 ± 0.06^B,d^
	CSO	1.70 ± 0^D,a^	4.15 ± 1.82^AD,b^	7.35 ± 0.28^C,c^	8.46 ± 0.96^A,d^
	SSO	2.38 ± 0.05^E,a^	14.10 ± 2.54^B,b^	16.78 ± 2.23^D,c^	17.73 ± 2.25^C,d^
p-AV	CO	5.69 ± 0.66^A,a^	36.44 ± 11.95^A,b^	73.26 ± 14.82^AC,c^	101.53 ± 22.22^A,d^
	SFO	6.18 ± 0.57^AB,a^	40.75 ± 9.61^A,b^	105.25 ± 7.47^B,c^	137.26 ± 4.31^B,d^
	SO	7.55 ± 0.81^BD,a^	41.44 ± 8.51^A,b^	81.78 ± 8.96^A,c^	106.40 ± 12.39^A,d^
	CSO	10.05 ± 0.46^C,a^	33.34 ± 1.14^A,b^	58.19 ± 3.33^C,c^	95.43 ± 12.45^A,d^
	SSO	8.88 ± 2.05^CD,a^	34.50 ± 8.56^A,b^	76.59 ± 13.93^A,c^	92.09 ± 11.30^A,c^
TOTOX	CO	7.34 ± 0.69^A,a^	41.44 ± 12.62^A,b^	78.87 ± 15.43^A,c^	112.02 ± 26.09^A,d^
	SFO	6.91 ± 0.67^A,a^	62.99 ± 10.53^B,b^	129.69 ± 1.43^B,c^	166.35 ± 3.31^B,d^
	SO	8.53 ± 0.80^A,a^	58.13 ± 8.37^B,b^	103.60 ± 8.67^C,c^	131.93 ± 12.49^A,d^
	CSO	13.47 ± 0.47^B,a^	41.64 ± 3.03^A,b^	72.90 ± 3.63^A,c^	112.36 ± 10.52^A,d^
	SSO	13.65 ± 1.94^B,a^	62.71 ± 4.54^B,b^	110.16 ± 17.57^C,c^	127.57 ± 11.36^A,c^
TPC	CO	6.33 ± 0.25^AB,a^	10.00 ± 0.54^AC,b^	12.50 ± 1.22^AB,c^	16.50 ± 1.44^A,d^
	SFO	6.75 ± 0.27^A,a^	10.58 ± 0.58^A,b^	15.41 ± 1.02^C,c^	18.83 ± 0.40^B,d^
	SO	6.08 ± 0.20^B,a^	8.83 ± 0.40^B,b^	10.25 ± 0.61^D,c^	15.83 ± 0.60^A,d^
	CSO	6.25 ± 0.27^B,a^	9.58 ± 0.37^BC,b^	11.41 ± 1.31^AD,c^	15.91 ± 0.37^A,d^
	SSO	6.45 ± 0.24^AB,a^	10.25 ± 0.52^AC,b^	13.91 ± 1.59^BC,c^	18.75 ± 0.52^B,d^
FFA	CO	0 ± 0^A,a^	0.31 ± 0.04^AC,b^	0.53 ± 0.12^AB,c^	0.86 ± 0.16^A,d^
	SFO	0 ± 0^A,a^	0.38 ± 0.04^C,b^	0.80 ± 0.10^B,c^	1.08 ± 0.09^B,d^
	SO	0 ± 0^A,a^	0.18 ± 0.04^B,b^	0.35 ± 0.05^A,c^	0.81 ± 0.07^A,d^
	CSO	0 ± 0^A,a^	0.28 ± 0.04^A,b^	0.45 ± 0.10^A,c^	0.83 ± 0.05^A,d^
	SSO	0 ± 0^A,a^	0.35 ± 0.05^AC,b^	0.68 ± 0.14^B,c^	1.06 ± 0.08^B,d^
TBARs	CO	1.07 ± 0.07^A,a^	2.93 ± 0.49^AB,b^	3.54 ± 0.68^AB,c^	4.68 ± 1.00^AB,d^
	SFO	0.58 ± 0.12^B,a^	2.31 ± 0.35^A,b^	3.33 ± 0.46^A,c^	3.70 ± 0.58^A,d^
	SO	0.91 ± 0.10^A,a^	3.24 ± 0.47^B,b^	3.92 ± 0.21^AB,c^	5.73 ± 1.52^B,d^
	CSO	1.33 ± 0.05^C,a^	3.24 ± 0.56^B,b^	4.24 ± 0.24^B,c^	5.44 ± 0.59^B,d^
	SSO	1.46 ± 0.16^C,a^	3.21 ± 0.30^B,b^	3.99 ± 0.36^AB,c^	4.93 ± 0.49^AB,d^
CD	CO	3.42 ± 0^A,a^	4.51 ± 0.46^A,b^	7.03 ± 1.34^A,c^	11.02 ± 1.12^A,d^
	SFO	1.65 ± 0.17^B,a^	4.52 ± 0.35^A,b^	13.40 ± 4.30^B,c^	16.01 ± 2.22^B,d^
	SO	5.27 ± 0.05^C,a^	8.15 ± 0.19^B,b^	11.33 ± 0.02^B,c^	14.10 ± 0.20^C,d^
	CSO	3.55 ± 0.02^A,a^	5.60 ± 0.12^C,b^	7.12 ± 0.19^A,c^	10.46 ± 0.22^A,d^
	SSO	3.47 ± 0.03^A,a^	7.88 ± 0.64^B,b^	13.29 ± 0.52^B,c^	16.89 ± 0.19^B,d^
CT	CO	2.05 ± 0^A,a^	2.39 ± 0.29^AC,b^	2.75 ± 0.41^A,c^	3.38 ± 0.28^A,d^
	SFO	1.50 ± 0.01^B,a^	2.00 ± 0.03^B,b^	3.55 ± 0.69^B,c^	4.26 ± 0.33^B,d^
	SO	1.29 ± 0.14^C,a^	2.04 ± 0.05^B,b^	2.80 ± 0.02^A,c^	3.25 ± 0.05^A,d^
	CSO	1.77 ± 0^D,a^	2.18 ± 0.04^AB,b^	2.44 ± 0.02^A,c^	3.31 ± 0.04^A,d^
	SSO	1.38 ± 0.03^C,a^	2.44 ± 0.06^C,b^	3.54 ± 0.06^B,c^	4.92 ± 0.63^C,d^

CO: corn oil; SFO: sunflower oil; SO: sesame oil; CSO: sesame-corn oil; SSO: sunflower-sesame oil. For each analysis, different small letters in each row show significant differences at level of *p* < 0.05. For each analysis, different capital letters in each column show significant differences at level of *p* < 0.05.

## Data Availability

Unfortunately, due to our university policy, the data are not available for public.

## Abbreviations

FFO: Sunflower oil
CO: Corn oil
SO: Sesame oil
SSO: Sunflower/sesame oil
CSO: Corn/sesame oil
GC-FID: Gas chromatography–flame ionization detection
DPPH: 2,2-Diphenyl-1-picrylhydrazyl
OSI: Oxidative stability index
RSD: Radical scavenging activity
PV: Peroxide value
p-AV: p-Anisidine value
TOTOX value: Total oxidation value
TPC: Total polar content
FFA: Free fatty acid
TBARS: Thiobarbituric acid-reactive substances
CD: Conjugated diene
CT: Conjugated triene
PUFA: Polyunsaturated fatty acid
MUFA: Monounsaturated fatty acid
SFA: Saturated fatty acid.
